# Impact of Melatonin Supplementation in Women with Unexplained Infertility Undergoing Fertility Treatment

**DOI:** 10.3390/antiox8090338

**Published:** 2019-08-23

**Authors:** Javier Espino, María Macedo, Graciela Lozano, Águeda Ortiz, Carmina Rodríguez, Ana B. Rodríguez, Ignacio Bejarano

**Affiliations:** 1 Department of Physiology (Neuroimmunophysiology and Chrononutrition Research Group), Faculty of Science, University of Extremadura, 06006 Badajoz, Spain; 2 Center for Human Assisted Reproduction of Extremadura (CERHA), 06010 Badajoz, Spain; 3 Department of Physiology and Pharmacology, Faculty of Medicine, University of Cantabria, 39011 Santander, Spain

**Keywords:** unexplained infertility, in vitro fertilization, oocyte quality, follicular fluid, melatonin, antioxidant

## Abstract

Unexplained infertility occurs when common causes for a couple’s inability to conceive have been excluded. Although origins of idiopathic infertility are still unclear, factors, such as an altered oxidative balance, are believed to be involved. Melatonin is an outstanding antioxidant reportedly present in the follicular fluid (FF), which has been suggested as a useful tool in the management of human fertility. Herein, we observed that intrafollicular concentrations of melatonin were blunted in women with unexplained infertility (UI), which was associated with a marked oxidative imbalance in UI patients’ FF. Based on these findings, this randomized pilot study was aimed at assessing whether exogenous melatonin ameliorated oxidative stress and improved in vitro fertilization (IVF) success rates in UI. Thus, 3 mg/day or 6 mg/day of melatonin were given to UI patients for a period spanning from the first appointment to control ovarian stimulation until the day of follicular puncture. Our results indicate that melatonin supplementation, irrespective of the two doses tested, ameliorated intrafollicular oxidative balance and oocyte quality in UI patients, and that this translated into a slight increase in the rate of pregnancies/live births. Therefore, although the indoleamine has shown therapeutic potential in this clinical setting, larger clinical trials in populations with different backgrounds are encouraged to corroborate the usefulness of melatonin.

## 1. Introduction

Infertility is considered as failure to conceive after a year or more of frequent unprotected sexual intercourse [1]. Infertility is said to be unexplained when common causes of infertility, such as absent ovulation and poor semen quality or tubal pathology, have been excluded after the completion of standard fertility tests [2]. Although causes of idiopathic infertility are currently unknown, factors, such as a perturbed oxidative balance, may affect women fertility. In fact, an overabundance of reactive oxygen species (ROS) in the follicular fluid (FF) of women with idiopathic infertility has been reported [3]. Other authors have found low levels of antioxidants in the FF of women undergoing evaluation for idiopathic infertility [4]. Besides, it is worth noting that embryo quality and oocyte maturation are negatively correlated with high levels of ROS in the FF of infertile women [5,6].

Melatonin is an indoleamine with low molecular weight that is synthesized by different cells and organs in the body [7]. In the reproductive system, melatonin and its synthetic machinery is present in the ovary and the placenta [8]. Indoleamine is acknowledged as a multitasking molecule that possesses remarkable biological functions, including regulation of biological rhythms [9], reproduction [10], and immune response [11,12], as well as anticancer [13] and metabolic effects [14]. More importantly, melatonin is an outstanding antioxidant that neutralizes oxidative stress by scavenging ROS and stimulating endogenous antioxidant enzymes [15,16].

The beneficial effects of melatonin supplementation on culture media [5,6,17,18], gametes [19,20,21], embryos [22,23,24], and luteal function [25,26] have suggested that indoleamine could be useful in the management of human fertility. In this sense, some clinical studies have recently tested the effect of oral supplementation of melatonin during ovarian stimulation on oocyte and embryo quality [5,27,28]. Nonetheless, the therapeutic potential of exogenous melatonin on unexplained infertility (UI) has not been explored yet. Therefore, this study was intended to test whether melatonin supplementation ameliorates the oxidative balance in the FF and oocyte quality of women with UI and whether this translates into enhanced success of in vitro fertilization (IVF) techniques.

## 2. Materials and Methods

### 2.1. Subjects

This was a pilot study carried out in 40 women who attended the Centre for Assisted Human Reproduction (CERHA, Badajoz, Spain), and was approved by the local ethics committees in accordance with the Declaration of Helsinki (#18002604). Informed consent was obtained from all the participants. Patients were excluded if they were < 18 years, active smokers, or were concurrently using other adjuvant therapies (e.g., Chinese herbs) (to avoid confounding factors since such herbs may contain high melatonin concentrations). Patients were also excluded if they had a history of autoimmune disorders/hypersensitivity to melatonin or its metabolites (to avoid potential side-effects) or were unable/were unwilling to comply with the study procedures. Patients and healthy volunteers were excluded if they had misused alcohol in the preceding three months.

### 2.2. Experimental Design

All women participating in the study were divided into 4 groups. Group 1 (control) was formed by healthy fertile women (*n* = 10) who had previously had at least one child (previous problems of fertility in the couple were due to male factor). Patients with UI (*n* = 30) were women subjected to a second IVF cycle who presented normal ovulation and any tubal pathology as ascertained by menstrual history and hysterosalpingogram, and their spouses were normospermic according to WHO criteria. Such patients were divided randomly into three groups of 10 women by using a computer-generated random number list: group 2, UI women who did not take melatonin; group 3, UI women who took a daily dose of 3 mg of melatonin; and group 4, UI women who took a daily dose of 6 mg of melatonin. In both cases, melatonin was taken one hour before going to sleep for a period spanning from the first appointment to control ovarian stimulation until the day of follicular puncture, i.e., for 40 days. Compliance with melatonin treatment was checked by capsule counting on the day of follicular puncture, and timing of melatonin dosing was self-reported in a patient compliance diary. Melatonin treatments comprised immediate-release melatonin formula (Guinama, Valencia, Spain) that was encapsulated in identical two-piece gelatine capsules (containing 3 mg or 6 mg melatonin) and dispensed in identical 50-capsule containers. These preparations were selected in light of previous studies demonstrating efficacy in individuals with infertility [5,21].

The baseline characteristics of the participants, including age, body mass index (BMI), and serum levels of follicle-stimulating hormone (FSH), luteinizing hormone (LH), and estradiol (E2), are presented in Table 1. Of note, there was no intergroup differences in any of the parameters analyzed. 

### 2.3. Ovarian Stimulation and Follicular Puncture

A standardized protocol with gonadotropin releasing-hormone (GnRH) antagonists was used for ovarian stimulation, as previously published [21]. After stimulation protocol, an average of 10 follicles were detected and aspirated by ultrasound-guided transvaginal needle aspiration.

### 2.4. Collection of Samples

First-void morning urines and follicular fluids (FF) were collected under basal conditions (groups 1 and 2) and after melatonin administration (groups 3 and 4), coinciding with the day of follicular puncture. FF was recovered from all follicles by transvaginal needle aspiration. All samples were protected from light and frozen at −80 °C until further analysis.

### 2.5. Embryo Classification

After sperm microinjection (ICSI) into the egg, the fertilization results were analyzed at 16 to 18 h after insemination (day + 1). The correctly fertilized oocytes (with 2 pronuclei and 2 polar bodies) were left in culture and their development was observed on day 2 and day 3, and transfer of a single fresh embryo was carried out approximately 48 or 72 h after the microinjection. Embryo quality was graded by blinded embryologists from “A” to “D” according to the embryo classification criteria proposed by ASEBIR [29]. Such classification is as follows: Grade A, blastomeres of equal size with <10% fragmentation (embryos of optimum quality and maximum implantation capacity); grade B, blastomeres of equal size with 11% to 25% fragmentation (embryos of good quality and high implantation capacity but not indicated for elective transfer of a single embryo); grade C, blastomeres of different size with 26% to 35% fragmentation (low quality embryos with a probability of medium implantation); grade D, which includes multinucleated embryos with > 35% fragmentation (lower quality embryos with a low implantation probability). For the present study, embryos with < 25% fragmentation (grade A or B) were considered as embryos suitable to be transferred.

### 2.6. Urinary 6-Sulfatoxymelatonin (aMT6s) Determination

Urinary 6-sulfatoxymelatonin (aMT6s), the major urinary metabolite of melatonin, was measured by means of an ELISA kit (IBL, Hamburg, Germany). Determinations were corrected for urinary creatinine by using Jaffe test [21] and therefore results are expressed as µg urinary aMT6s: µg urinary creatinine.

### 2.7. Quantification of Melatonin

Intrafollicular melatonin levels were determined by means of a commercial enzyme-linked immunosorbent assay (ELISA) kit (IBL). Concentrations of melatonin were corrected for the protein content of the FF by using Bradford method. Results are expressed as pg melatonin/mg protein.

### 2.8. Total Antioxidant Capacity (TAC) Assay

Total antioxidant capacity (TAC) was evaluated in the FF as previously described [21]. The capacity of the antioxidants in the sample was quantified as μM Trolox equivalents. Determinations were corrected for the protein content of the FF by using the Bradford method and therefore results are expressed as μM Trolox/mg protein.

### 2.9. Estimation of Superoxide Dismutase (SOD) Activity

To determine superoxide dismutase (SOD) activity in the FF, a colorimetric assay kit (Cayman Chemicals, Ann Arbor, MI, USA) was used. Determinations were corrected for the protein content of the FF by using the Bradford method. The final SOD activity levels are therefore expressed as units of enzymatic activity per mg of protein contained in the samples (U/mg protein).

### 2.10. Measurement of Lipid Peroxidation (LPO)

Intrafollicular concentrations of malondialdehyde (MDA), a product of the decomposition of polyunsaturated fatty acid peroxides, were measured as an indicator of lipid peroxidation (LPO). To do so, a colorimetric assay kit (Oxford Biomedical Research, Rochester Hills, MI, USA) was used. Determinations were corrected for the protein content of the FF by using the Bradford method. The LPO index is expressed as µM MDA per mg of protein contained in the samples (µM MDA/mg protein).

### 2.11. DNA Oxidative Damage Assay

DNA oxidative damage was detected by determining intrafollicular concentrations of 8-hydroxy-2′-deoxyguanosine (8-OHdG) by means of an ELISA kit (Cayman Chemicals). Determinations were corrected for the protein content of the FF by using the Bradford method and results are expressed as ng 8-OHdG/mg protein.

### 2.12. Statistical Analysis

Data are expressed as means ± standard deviation (SD) of the number of determinations. For multiple comparisons of non-categorical variables, one-way analysis of variance followed by Tukey’s tests was used. The Chi-squared test was used to determine intergroup differences for the observed frequencies of mature oocytes, fertilized oocytes, and clinical pregnancies per initiated cycle. Pearson’s correlation by multiple regression of different parameters of intrafollicular oxidative balance with melatonin levels in FF was also tested. *p* < 0.05 was considered a statistically significant difference.

## 3. Results

### 3.1. Analysis of Melatonin Concentrations in Urine and Follicular Fluid (FF)

**Figure 1:**
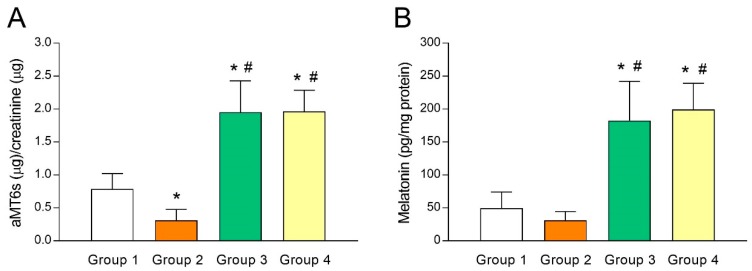
Melatonin levels in urine and follicular fluid of patients with unexplained infertility and the effect of melatonin supplementation. Urinary levels of 6-sulfatoxymelatonin (aMT6s) (**A**) and intrafollicular concentrations of melatonin (**B**) in fertile women (group 1) and unexplained infertility (UI) patients untreated (group 2) or treated with 3 mg/day (group 3) or 6 mg/day (group 4) of melatonin. Values represent means ± SD of 10 individuals. * *p* < 0.05 vs. group 1. ^#^ *p* < 0.05 vs. group 2.

As melatonin concentrations in FF has been shown to predict IVF outcomes and ovarian reserve [30,31], putative differences in melatonin levels of fertile women (group 1) and UI patients (group 2) were analyzed. For this purpose, we first gauged urine aMT6s (indicator of systemic melatonin) levels and observed a lower aMT6s concentration (*p* < 0.05) in group 2 compared to group 1 (Figure 1A). The same trend held true when melatonin concentrations were measured in FF (Figure 1B), although the difference was not statistically significant. These results may reflect an association between low melatonin concentrations and poor fertility and are in line with the predictive potential of melatonin for IVF outcomes [30,31]. As expected, aMT6s concentrations increased by several folds (*p* < 0.05) in those UI patients who were given 3 mg (group 3) or 6 mg melatonin/day (group 4) for 40 days (Figure 1A), indicating that a single daily dose of melatonin was enough to raise systemic concentrations of the indoleamine. Consequently, such patients presented a substantial (*p* < 0.05) rise in intrafollicular concentrations of melatonin (Figure 1B).

### 3.2. Intrafollicular Oxidative Balance in UI Patients and Effect of Melatonin Administration

**Figure 2:**
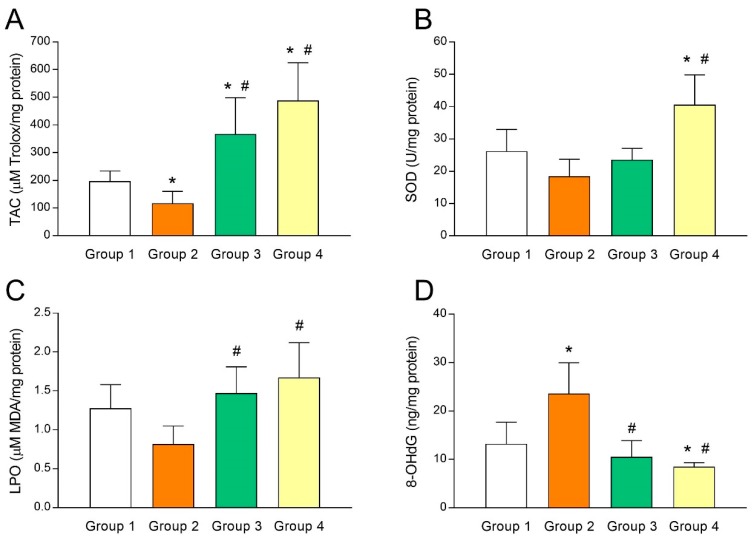
Altered intrafollicular oxidative balance in patients with unexplained infertility is countered by melatonin administration. (**A**) Total antioxidant capacity (TAC), (**B**) superoxide dismutase (SOD) activity, (**C**) lipid peroxidation (LPO), and (**D**) 8-hydroxy-2’-deoxyguanosine (8-OHdG) concentrations in the follicular fluids of fertile women (group 1) and UI patients untreated (group 2) or treated with 3 mg/day (group 3) or 6 mg/day (group 4) of melatonin. Values represent means ± SD of 10 individuals. * *p* < 0.05 vs. group 1. ^#^ *p* < 0.05 vs. group 2.

**Figure 3:**
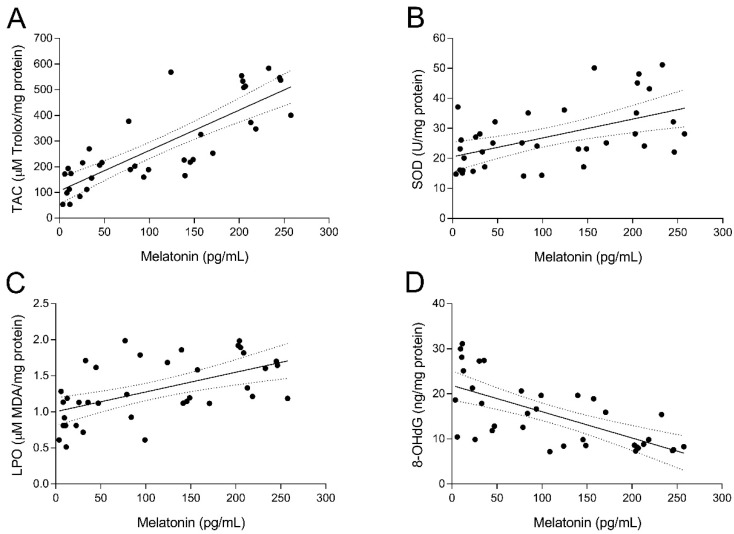
Relation between the intrafollicular concentrations of melatonin and different biomarkers of oxidative balance. Pearson’s correlation between intrafollicular concentrations of melatonin and intrafollicular total antioxidant capacity (TAC) (**A**), superoxide dismutase (SOD) activity (**B**), lipid peroxidation (LPO) (**C**), and 8-hydroxy-2′-deoxyguanosine (8-OHdG) concentrations (**D**).

To study the relationship between UI and intrafollicular oxidative stress, several biomarkers of oxidative balance were determined in the FF sampled at oocyte retrieval in both fertile women and UI patients. As shown in Figure 2A, TAC levels in FF of fertile women (group 1) were significantly higher (*p* < 0.05) than those of UI patients (group 2). Of note, administration of melatonin to UI patients caused a marked (*p* < 0.05) increase of intrafollicular TAC levels independently from the dose of melatonin (group 3 and group 4; Figure 2A). Moreover, TAC levels in the FF were significantly and positively correlated with intrafollicular concentrations of melatonin (*y* = 1.572*x* + 106.1; *r* = 0.8202; *p* < 0.05) (Figure 3A). In the same line, intrafollicular SOD activity was shown to be partly reduced in UI patients (group 2) compared to fertile women (group 1) (Figure 2B). Importantly, melatonin supplementation significantly enhanced (*p* < 0.05) intrafollicular SOD activity in those UI patients receiving the dose of 6 mg/day (group 4) but not in those taking 3 mg/day (group 3) (Figure 2B). Like TAC levels, SOD activity in the FF was positively correlated with intrafollicular concentrations of melatonin (*y* = 0.0628*x* + 20.53; *r* = 0.5217; *p* < 0.05) (Figure 3B).

The oxidative balance in UI patients was further examined by analyzing concentrations of intrafollicular LPO products. Paradoxically, UI patients (group 2) exhibited slightly reduced levels of intrafollicular LPO products compared to fertile women (group 1) (Figure 2C). Administration of melatonin restored concentrations of LPO products in the FF of UI patients to levels found in fertile women (*p* < 0.05), irrespective of melatonin dose (group 3 and group 4; Figure 2C). Additionally, intrafollicular LPO products were positively correlated with intrafollicular concentrations of melatonin (*y* = 0.0027*x* + 1.004; *r* = 0.5536; *p* < 0.05) (Figure 3C).

Finally, concentrations of 8-OHdG as a biomarker of oxidative stress were also determined in the FF of both fertile women and UI patients. Intrafollicular 8-OHdG concentrations in UI patients were dramatically (*p* < 0.05) higher than those in fertile women (Figure 2D). Remarkably, melatonin supplementation significantly (*p* < 0.05) decreased intrafollicular 8-OHdG levels in those UI patients who were given 3 mg (group 3) or 6 mg melatonin/day (group 4) (Figure 2D). Besides, intrafollicular concentrations of 8-OHdG were significantly and negatively correlated with intrafollicular concentrations of melatonin (*y* = –0.0582*x* + 21.87; *r* = –0.6682; *p* < 0.05) (Figure 3D).

### 3.3. Clinical Outcomes of Melatonin Supplementation in IVF-Embryo Transfer for UI Patients

To test the clinical usefulness of melatonin administration in the context of UI, the effect of melatonin treatment on both in vitro oocyte quality and clinical outcomes of IVF embryo transfer for UI patients was examined. A total of 86 (8.7 ± 1.5) and 68 (6.8 ± 1.2) oocytes were retrieved in group 1 (fertile women) and group 2 (UI patients), respectively (Table 2). While the maturation, fertilization, and embryonic development in UI patients were similar to those in fertile women, the pregnancy rates in fertile women were higher (50.0% (5/10)) than that of UI patients (20.0% (2/10)) (Table 2). Importantly, administration of melatonin to UI patients significantly (*p* < 0.05) enhanced the number of oocytes retrieved, irrespective of the dose of melatonin (10.0 ± 1.7 and 9.5 ± 2.1 in group 3 and group 4, respectively; Table 2). The proportion of mature oocytes (83.6% in group 3), the percentage of fertilized oocytes (67.4% and 63.7% in group 3 and group 4, respectively), and the average number of transferable embryos (5.1 ± 1.1 and 4.6 ± 0.8 in group 3 and group 4, respectively) were significantly (*p* < 0.05) higher in UI patients treated with melatonin (Table 2). Consequently, the number of pregnancies per embryo transfer in those groups treated with melatonin were relatively higher (30.0% (3/10)) compared to those found in UI patients (Table 2).

## 4. Discussion

Herein, we showed that women with UI present a marked oxidative imbalance in their FF (e.g., low TAC levels or high 8-OHdG concentrations), which may influence the poor quality of their oocytes and therefore, may be related to the low success rate of ART (20%) observed in this group of patients. More importantly and in line with the importance of melatonin in oocyte maturation and embryonic development [32], reduced concentrations of melatonin were found both at the systemic level and in the FF of women with UI, with the melatonin levels being correlated with the different parameters of oxidative balance assessed in their FF. Additionally, we provided evidence that melatonin supplementation: (i) Positively impacted on the oxidative balance in the FF of women with UI since it is capable of restoring concentrations of diverse markers of oxidative status to levels found in fertile women; (ii) improved oocyte quality and, consequently, the amount of transferable embryos in UI patients; and (iii) slightly enhanced the success of ART, which increased up to 30% in the groups of women with UI treated with melatonin.

Women with UI enrolled in this study presented low melatonin concentrations along with limited antioxidant levels (TAC assay) in their FF. These findings agree with previous research reporting low levels of TAC in women undergoing evaluation for UI [4] and women with polycystic ovarian syndrome (PCOS) [33]. Besides, a direct relationship has been established between decreased TAC and poor oocyte and embryo quality and a low fertilization rate [34], which is in good agreement with our results in UI patients. Similarly, we observed that intrafollicular SOD activity found in untreated infertile patients was moderately lower in comparison with that of fertile women. Again, our findings fit into earlier studies indicating decreased SOD activity in the FF of women with PCOS [35]. Interestingly, we showed that melatonin supplementation reversed both reduced TAC levels and low SOD activity in UI patients, the dose of 6 mg melatonin/day being especially effective. The stimulating effect of the indoleamine on antioxidant enzymes, such as SOD, is well known and has already been described in other tissues, such as the liver, kidney, or brain [36,37].

Despite the fact that elevated concentrations of lipid peroxides have been associated with female reproductive diseases [33,38], it seems that a certain threshold level of LPO, resulting from an obligatory minimal metabolic activity within the preovulatory follicle, is necessary to establish a pregnancy [34,39]. In fact, intrafollicular LPO levels were found to be positively correlated with the outcomes of ART [39]. This apparent controversy could be explained because a certain amount of oxygen (> 5% O_2_) seems critical to affect the oocyte’s capacity to mature to metaphase II, to form a functional spindle, and to align chromosomes correctly [40]. In this sense, slightly lower intrafollicular LPO levels were found in UI patients enrolled in the present study, which could have negatively affected patients’ oocyte quality and hence, their reproductive capacity. Additionally, we observed that supplementation with 3 or 6 mg/day of melatonin resulted in an increase in intrafollicular LPO levels of UI patients, thereby reaching values similar to those of fertile women. These findings, although apparently paradoxical, confirm that melatonin restored the altered intrafollicular oxidative balance of infertile patients, turning their FF into a fertile-like microenvironment.

Excessive ROS cause both single- and double-strand DNA breaks, thus producing mutations in both nuclear and mitochondrial DNA [41]. Oxidative damage to DNA can be quantified by measuring 8-OHdG concentrations, and high levels of this metabolite are also indicative of poor embryo quality and low pregnancy rates [42]. In this line, our results showed that untreated UI patients had higher intrafollicular levels of 8-OHdG than those observed in fertile women. More importantly, infertile patients benefited from supplementation with the indoleamine since either 3 or 6 mg/day of melatonin dramatically lessened follicular DNA oxidative damage, which is in accordance with previous studies [5].

Finally, it was shown that the improvement in the intrafollicular oxidative balance of UI patients due to melatonin supplementation resulted in an enhancement of both oocyte/embryo quality and pregnancy rates. Particularly, our results indicate that the administration of the two doses of melatonin tested produced a considerable increase in the number of oocytes retrieved, in the maturation rate, and in the fertilization rate of patients with idiopathic infertility. Even though embryo quality was not significantly improved, the availability of a higher proportion of transferable embryos due to the increase in oocyte fertilization rates led to a rise in the pregnancy rate per embryo transfer. In this sense, several authors have found that melatonin administration ameliorated embryo quality and fertilization rates, but no differences were observed in other parameters, such as maturation rates or blastocyst rates [5,24,43].

## 5. Conclusions

To sum up, we provided evidence that intrafollicular concentrations of melatonin, which has previously been suggested as a biomarker to predict IVF outcomes and ovarian reserve [30,31], were blunted in UI patients. Such reduced melatonin levels were also associated with a marked oxidative imbalance in patients’ FF. Importantly, melatonin supplementation re-balanced the intrafollicular oxidative status, improved oocyte quality, and slightly enhanced IVF success rates in UI patients. As both doses of the indoleamine tested were effective, this suggests that the lower dose (3 mg/day) is sufficient to ameliorate the harmful microenvironment in the FF of such patients and hence, their oocyte quality. Although relevant, these findings should be treated with caution because of the limited sample size and the lack of blinding of group allocation. Before a definite recommendation is made on the use of melatonin in women with UI, larger clinical studies in populations with different backgrounds need to be performed to confirm the therapeutic potential of the indoleamine in this clinical setting.

## Figures and Tables

**Table 1:** Baseline features of participants enrolled in the study.

Variables	Group 1	Group 2	Group 3	Group 4	*p* Value
**Age (years)**	36.27 ± 2.08	35.93 ± 3.20	34.73 ± 3.03	36.22 ± 2.71	0.577
**BMI (kg/m^2^)**	24.07 ± 3.29	23.83 ± 3.53	23.42 ± 2.88	23.66 ± 3.40	0.975
**bE_2_ (pg/mL)**	57.99 ± 22.04	59.21 ± 29.17	56.37 ± 22.19	56.50 ± 26.63	0.993
**bLH (IU/mL)**	6.49 ± 1.94	6.60 ± 3.29	7.32 ± 3.11	5.91 ± 2.85	0.744
**bFSH (IU/mL)**	9.42 ± 3.53	10.21 ± 4.68	9.28 ± 3.40	9.62 ± 3.33	0.949

Data represent the mean ± SD (standard deviation) of 10 patients. BMI: body mass index; E_2_: basal estradiol; bLH: basal luteinizing hormone; bFSH: basal follicle-stimulating hormone.

**Table 2:** Clinical outcomes of in vitro fertilization (IVF)-embryo transfer in fertile women and patients with unexplained infertility treated with different dosages of melatonin.

Variables	Group 1	Group 2	Group 3	Group 4
Average no. of oocytes retrieved (95% CI)	8.7 (5.9–11.5)	6.8 (4.4–9.2)	10.0 ^#^ (6.7–13.3)	9.5 ^#^ (5.8–13.5)
% of mature oocytes (k/n)	81.9 (86/105)	70.6 (48/68)	83.6 ^#^ (92/110)	76.2 (80/105)
% of fertilized oocytes (k/n)	51.1 (44/86)	47.9 (23/48)	67.4 ^#^ (62/92)	63.7 ^#^ (51/80)
Average no. of transferable embryos (95% CI)	2.3 (0.5–4.0)	2.0 (0.4–3.6)	5.1 *^#^ (2.8–7.4)	4.6 *^#^ (2.8–6.3)
% of clinical pregnancies/initiated cycle (k/n)	50.0 (5/10)	20.0 (2/10)	30.0 (3/10)	30.0 (3/10)
no. of full-term pregnancies	5	2	3	3

* *p* < 0.05 *vs*. group 1. ^#^ *p* < 0.05 vs. group 2. CI: confidence interval; k: number of observations that are of interest; n: total number of observations.
